# Intravitreal ocriplasmin for the treatment of vitreomacular traction and macular hole- A study of efficacy and safety based on NICE guidance

**DOI:** 10.1371/journal.pone.0197072

**Published:** 2018-05-16

**Authors:** Mahiul M. K. Muqit, Robin Hamilton, Jason Ho, Sally Tucker, Helen Buck

**Affiliations:** 1 Department of Vitreoretinal Surgery, Moorfields Eye Hospital, London, United Kingdom; 2 Department of Medical Retina, Moorfields Eye Hospital, London, United Kingdom; 3 SJT Focus Limited, Harpenden, United Kingdom; Massachusetts Eye & Ear Infirmary, Harvard Medical School, UNITED STATES

## Abstract

**Purpose:**

To evaluate the real world clinical outcomes of intravitreal ocriplasmin in patients with vitreomacular traction (VMT) with and without full thickness macular holes (FTMH) treated according to NICE guidance.

**Methods:**

Retrospective observational case series of 25 patients treated with a single intravitreal ocriplasmin injection between December 2013 and December 2015. Best corrected visual acuity and optical coherence tomography exams were performed to determine visual outcomes and anatomical VMT release and FTMH closure over time. Two patient groups were identified: ocular macular co-morbidity (OCM) and no OCM (nOCM), with follow-up at 4, 12, and 24 weeks.

**Results:**

Twenty-five patients were identified that included 19 patients with VMT, and 6 patients with VMT plus FTMH. In the nOCM group of 22 patients, the release rate of VMT was 44%, 63%, and 69% at 4, 12 and 24 weeks respectively. In the “real-world” OCM group of 25 patients, the VMT release rate was 37%, 53%, and 58% at the same time-points. In both groups, the FTMH closure rate was 33%, 50%, and 67% at 4, 12, and 24 weeks. At mean follow-up of 30 weeks in the VMT group with nOCM, the mean LogMAR VA improved significantly from 0.44 to 0.28 (p = 0.0068, paired t-test). Three were no serious adverse events.

**Conclusions:**

This study reports improved efficacy of intravitreal ocriplasmin for both VMT and FTMH, and is more favourable in patients with no ocular co-morbidity. We highlight the importance of careful patient selection and structured standard of care pathways to identify patients who will benefit from the positive visual and anatomical effects of intravitreal ocriplasmin.

## Introduction

With age the vitreous body shrinks and undergoes a process of liquefaction (synchisis) [1,2]. The vitreous becomes filled with fluid-filled lacunae that leads to reduction in the vitreous gel volume. This results in the forward movement of the vitreous as it separates from the retinal surface, and there is collapse of the vitreous gel (syneresis), which can cause it to detach from the central macula and optic disc (posterior vitreous detachment, PVD). The posterior vitreous gel and retina form the vitreoretinal interface, with vitreous cortex fibres inserting into the internal limiting membrane of the macula and retina (2). At this interface, there are adhesion molecules that include fibronectin, heparan sulphate, and laminin proteoglycans.

During the initial stages of PVD in some individuals, adhesions remain between the vitreous body and retina. The vitreous gel itself is most firmly attached to the retina at the vitreous base, optic disc, and fovea, and along the major retinal blood vessels. When these vitreoretinal adhesions involve the macula, this is referred to as vitreomacular adhesion (VMA) based on optical coherence tomography (OCT) grading [3–5]. VMA is generally an asymptomatic condition that usually resolves spontaneously. In cases where VMA persists, the VMA may progress to vitreomacular traction (VMT) [6], a condition of the vitreoretinal interface, resulting from abnormally strong adhesions between the vitreous gel and the central fovea. In VMT, typical symptoms include distortion, metamorphopsia, micropsia and blurred vision. VMT is a progressive vitreoretinal interface disorder that can result in the development of a macular hole (MH). The MH can be either lamellar hole or a full-thickness macular hole (FTMH), and it is the FTMH that can result in significant visual loss and distortion.

To date, the only treatment option for progressive, symptomatic VMT is vitrectomy surgery commonly combined with cataract surgery, which removes the adhesions and traction between the vitreous and the macula. For patients with VMT, the prospect of vitrectomy surgery can be daunting and patients are often apprehensive regarding any potential surgical risks associated with vitrectomy and visual recovery is not guaranteed [7], with there being a possibility of further visual loss and moderate visual gains [8]. Vitrectomy is now limited to those individuals suffering from VMT where the condition had progressed with significant visual loss.

In 2013, the National Institute for Health and Care Excellence (NICE) approved intravitreal ocriplasmin (Jetrea^TM^, Alcon Laboratories, UK, Limited) [9], for patients with either VMT or VMT with a FTMH in the United Kingdom. This new treatment intervention involves the administration of an intravitreal molecule called ocriplasmin that is a proteolytic enzyme that breaks down the fibronectin and laminin at the pathological vitreoretinal interface [10] often referred to as a “chemical vitrectomy” or “pharmacological vitreolysis”. Its efficacy in the treatment of VMT has been demonstrated to be significantly better than that observed in placebo patients [11]. The clinical risks of this intervention are the recognised intravitreal injection-related risks and the following rare ocriplasmin drug-related risks that may include lens subluxation or phacodonesis, dyschromatopsia, reversible electroretinogram changes, self-limiting ellipsoid layer OCT changes, retinal tear or retinal detachment, and reduction in visual acuity.

NICE guidance recommends that intravitreal ocriplasmin treatment is only considered for treating VMT in patients with no evidence of an epiretinal membrane (ERM), they have a FTMH with a diameter up to 400 micrometers (μm) and/or they have severe symptoms. The NICE guidelines recommend careful evaluation of patients with VMT that meet these criteria, in order to increase the likelihood for success with ocriplasmin and maximise patient benefit. Indeed a recent study conducted by Willekens *et* al demonstrated the importance of careful patient selection to improve the efficacy of ocriplasmin in patients with VMT [12]. Optical coherence tomography (OCT) allows clinicians to examine the retina and the vitreoretinal interface non-invasively to determine the presence of VMA or VMT, as per the classifications provided by the vitreomacular traction study group [3]. It further allows confirmation of any ERM presence and the evaluation of FTMH.

The primary aim of this study is to look at the clinical effects and adverse effects for intravitreal ocriplasmin in patients with VMT and FTMH according to the NICE guidance. With the introduction of new NICE guidance, it is important to undertake clinical audit of the real-world clinical use to evaluate clinical practice and develop local treatment pathways.

## Materials and methods

### Audit data collection

This is a retrospective observational case series of consecutive patients who underwent a single intravitreal ocriplasmin injection at Moorfields Eye Hospital (MEH) between December 2013 and December 2015. Information collected for the study included age, gender, indication for treatment, best-corrected visual acuity (BCVA) using logMAR, clinical efficacy, outcomes, and complications following ocriplasmin. Data was also collected for patients who received subsequent surgery management. The audit included patients undergoing treatment with ocriplasmin who were under the care of seven consultants in three hospital locations (City Road, Croydon, and St Georges). Data was collated from the Moorfields High Cost pharmacy database, by review of the Hospital “Open Eyes” electronic database, and by case record review. Safety data was extracted from the events reported to Alcon Laboratories (UK) Limited as per the adverse event reporting system.

The study adhered to the tenets of the Declaration of Helsinki and was approved by the Moorfields Eye Hospital Audit Committee. The data was fully anonymized before access, and the Moorfields committee waived the requirement for informed consent according to the standard operating procedures for retrospective auditing. The images used in the article were fully anonymized and patient consent was not required according to institutional guidelines.

### Patient standard of care

**Fig 1:**
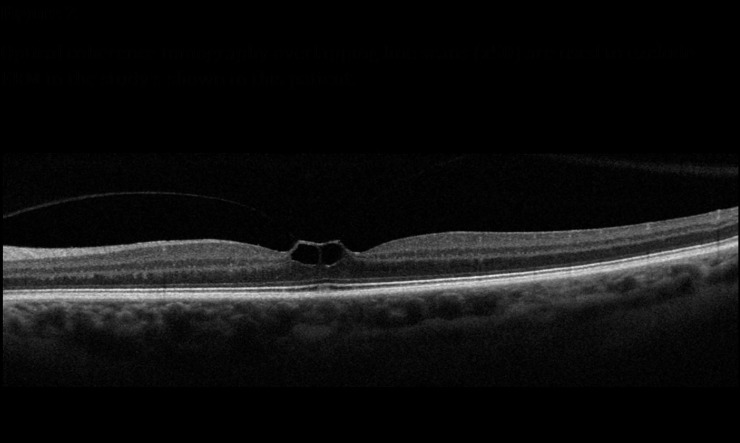
Optical coherence tomography scan of vitreomacular traction. Optical coherence tomography overlapping (x50) line scans are used to exclude ERM.

As part of standard of care, all patients underwent a complete ophthalmological examination, including slit-lamp examination, BCVA measurement using an ETDRS (LogMAR) chart and OCT. All patients had VMT or VMT associated with FTMH, as confirmed by OCT review (Fig 1). Patients were eligible for possible ocriplasmin treatment according to the NICE guidelines [9] i.e.:

OCT was used to confirm adherence to NICE guidelines for treatment with ocriplasmin.

- ERM was not present (see Fig 1B) and
- Stage II FTMH with a diameter of 400μm or less and/or
- Presence of severe symptoms.

There were no ocular or patient-related exclusion criteria, and only patients that had minimum follow-up of 28 days following ocriplasmin injection were included.

### Ocriplasmin administration and follow-up

Treatment was administered according to NICE guidelines, which comprised a single intravitreal injection at a dose of 0.125mg in 0.1ml. All intravitreal injections were performed under sterile conditions, according to the local standard procedures. The preferred injection technique was the hub needle technique with moderate injection velocity, and patients lay flat for 5 minutes post-injection to allow gravitational dispersion of the ocriplasmin to the macula area. Patients were observed for 30 minutes after injection and then discharged. Routine follow-up visits were scheduled for 1 week and 4 weeks following injection, with most patients being followed for up to 30 weeks. Assessments at follow-up included BCVA measurement and OCT examination. Adverse events were also recorded and reported as per the reporting requirements from the manufacturer.

### Clinical outcomes

The primary outcome for this study was the proportion of patients experiencing non-surgical resolution of the VMT and closure of the FTMH at four weeks following a single ocriplasmin injection. Secondary outcomes included visual acuity outcomes at 4, 12 and 24 weeks. We evaluated the ocular characteristics that included duration of symptoms, pseudophakic versus phakic, co-existing retinal comorbidities, and the outcomes of ocriplasmin failures that underwent subsequent vitrectomy surgery and cataract surgery. Data were grouped for patients with no ocular macular co-morbidity (nOCM), and patients with a confirmed diagnosis of associated ocular macular co-morbidity (OCM). The real world group was designated as the OCM group, as the doctors would decide to include patients with other macular co-mordidity based on clinical grounds. The published MIVI clinical trial did not include patients with other macular co-morbidity hence our decision to separate the two groups. In this way, we reported the total groups with OM and without OM, and this would show the effects of associated OM on final clinical outcomes. The data were entered onto an electronic audit database and summarised.

### Statistical methods

A Fischer’s exact test was used to compare patients that had a VMT release and those who did not. Paired t-test was used to compare pre-operative and post operative VA. A p-value less than 0.05 was defined as statistically significant, all analyses were performed using Prism version 6.0 (GraphPad Software, CA, USA).

## Results

### Baseline characteristics

Tables 1 and 2 present the clinical characteristics of the treated patients at baseline. Twenty-five patients in total were included in this study. Mean age was 71 years, with a higher proportion of female patients (17 of the 25 patients). Mean (range) duration of symptoms was 28 (7–156) weeks. The majority of VMT patients were referred through the care pathway as routine referrals. Patients with FTMH were referred more rapidly to the specialist clinics. The mean baseline LogMAR VA was 0.52 (6/20). Twenty-two patients had nOCM, 16 patients had VMT and 6 patients had VMT plus FTMH. Mean (range) pre-operative VMT width as measured on OCT was 536μm (71–816) and mean (range) pre-operative FTMH size was 250μm(159–537). In total, three patients had OCM that included diabetic macular edema, macular telangiectasia, and age-related macular degeneration. The three patients had VMT only and were all phakic. Patients with OCM had a longer duration of symptoms (mean duration 28 weeks versus 23 weeks)**,** which could be as a result of longer time period to diagnosis/referral.

**Table 1:** Baseline demographics and ocular characteristics.

	Real World(n = 25)	No OCM(n = 22)
Mean Age (years)	71	71
Pseudophakic: phakic	4:21	4:18
Gender Male:Female	8:17	6:16
Mean (range) duration of symptoms weeks	28 (6–156)	23 (6–80)
Mean pre-op BCVA LogMAR	0.52 (6/20)	0.52 (6/20)
Pre-op Mean (range) VMT microns	536 (71–816)	536 (71–816)
Pre-op Mean (range) FTMH microns	250 (159–357)	250 (159–357)

OCM, ocular comorbidity; BCVA, best-corrected visual acuity; VMT, vitreomacular traction; FTMH, full-thickness macular hole.

**Table 2:** Baseline characteristics of individual patients and anatomical resolution of VMT at 4, 12 and 24 weeks.

	Age	Gender	Concomitant Ocular disease	Lens status	Presence of ERM	VMT size (um)	FTMH size (um)	VMT resolution (weeks)		
								4	12	24
1	82	M		phakic	N	697	N		Y	Y
2	75	F	macular telangiectasia type 1	phakic	N	380	N		VMT stable	
3	78	F	glaucoma	phakic	N	598	N			
4	52	F		phakic	N	506	N	Y		Y
5	64	M	age-related macular degeneration	phakic	N	552	N			
6	82	F		pseudophakic	N	71	N		Y	Y
7	67	M	chronic diabetic macular edema	phakic	N	451	N		VMT, DME	
8	60	F		phakic	N		357	Y	Y	Y
9	71	M	glaucoma	phakic	N	207	N	Y		Y
10	78	F	Cataract	pseudophakic	N	505	N		Y	Y
11	81	F		pseudophakic	N	816	N			
12	71	M		phakic	N		159	Y		Y
13	64	F		phakic	N		322			
14	53	F		phakic	N	255	N			
15	83	M		phakic	N	560	N			
16	65	F	Cataract	PHAKIC	N	486	N	Y		Y
17	79	F	Cataract	phakic	N	807	N			
18	65	F	Cataract	phakic	N	726	N	Y		Y
19	79	F		pseudophakic	N	315	N	Y	Y	Y
20	78	F		phakic	N	598	N	Y	Y	Y
21	85	M		phakic	N		170		Y	Y
22	51	F	High myopia	PHAKIC	N		196			
23	67	M	Cataract	phakic	N		300	Y		Y
24	76	F	Cataract	phakic	N	678	N	Y		Y
25	54	F		phakic		761	N	Y		

ERM, epiretinal membrane; VMT, vitreomacular traction; FTMH, full-thickness macular hole; DME, diabetic macular edema.

### VMT resolution

**Fig 2:**
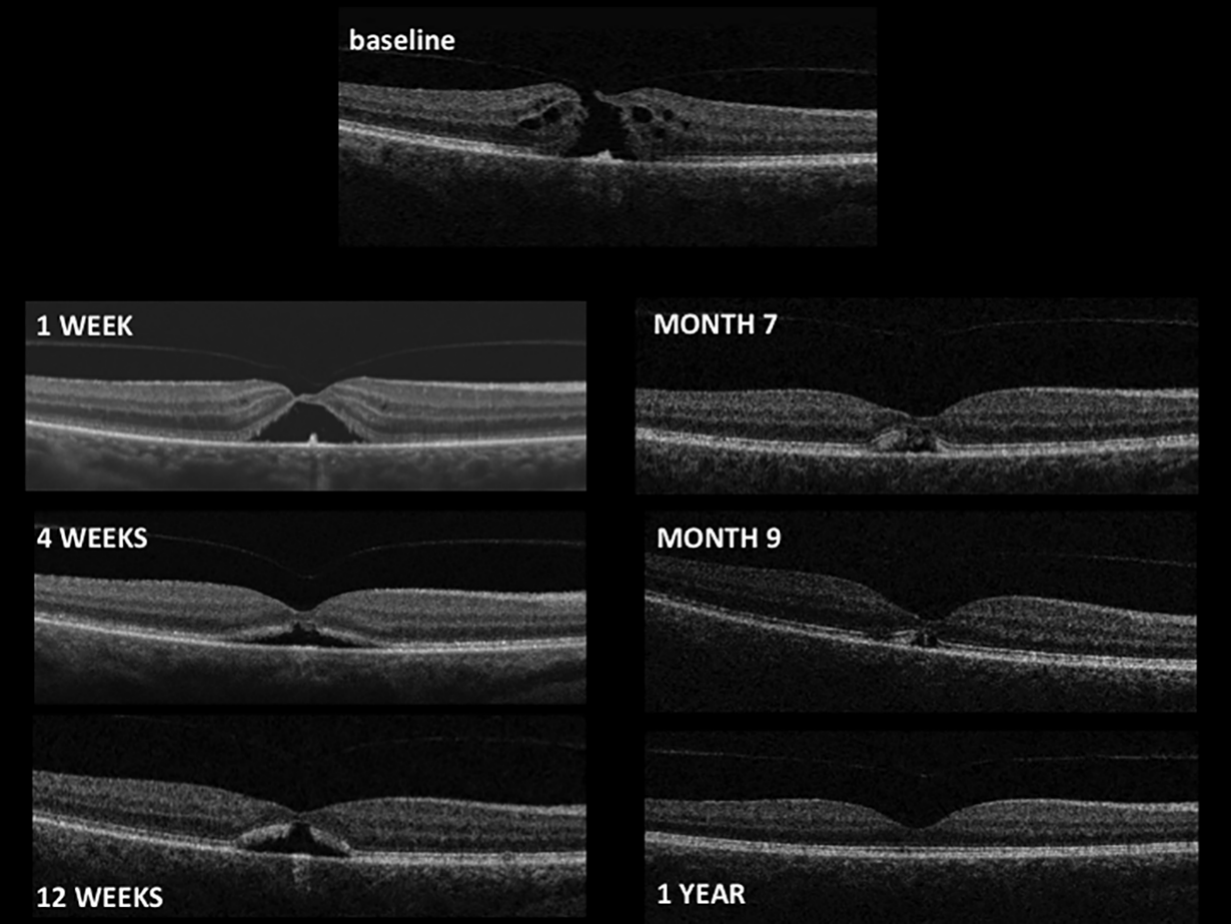
Case study of VMT and macular hole. This 60 year old female patient was diagnosed with right VMT and FTMH (357 microns), she had suffered for 2–3 months with blurred and distorted vision and was very symptomatic, the absence of ERM was confirmed. Vision prior to ocriplasmin injection was R 6/36, L 6/18. The patient was treated with ocriplasmin according to standard MEH protocols and followed up as standard of care. FTMH closed after one week. Vision at Month 7 was 6/24.

**Fig 3:**
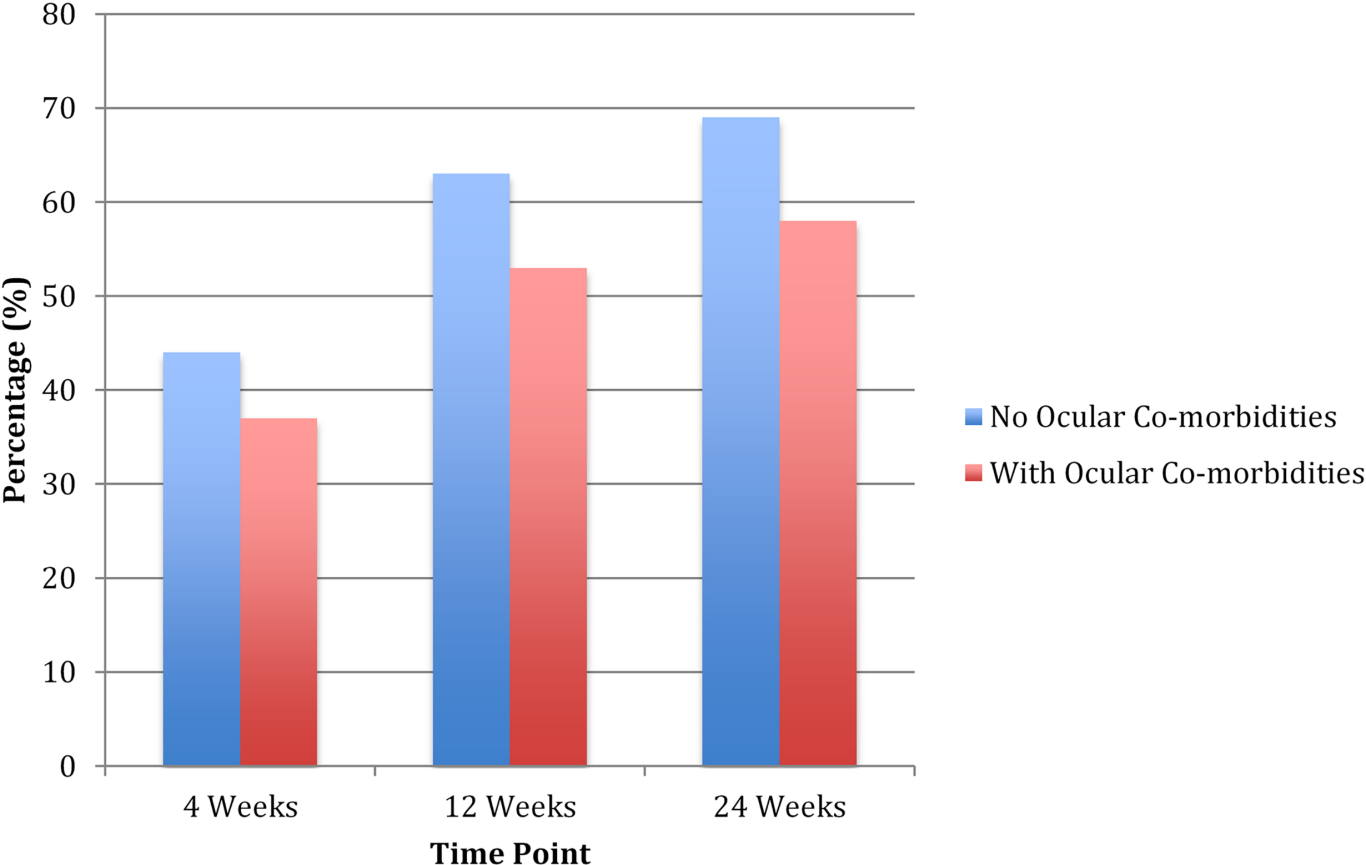
Resolution of vitreomacular traction. The VMT resolution rates at 4, 12 and 24 weeks following a single intravitreal injection of ocriplasmin (0.125 mg in 0.1ml). VMT was diagnosed by OCT as per the classifications provided by the VMT study group.

The primary efficacy endpoint for the audit was the non-surgical resolution of the VMT at 4 weeks (Figs 2 and 3). In the nOCM patients group, the resolution rate for the VMT was 44%. In the OCM patient group, the VMT success rate was reduced to 37% at 4 weeks. The success rates increased at 12 weeks (63%) and at 24 weeks (69%) in the nOCM group. Similarly, there was increased success in the OCM group with resolution rates of 53% and 58% at 12 and 24 weeks respectively (Table 3).

**Table 3:** Anatomical resolution of VMT at 4, 12 and 24 weeks.

	Baseline	4 Weeks		12 Weeks		24 Weeks	
**No OCM**		Res	Not res	Res	Not res	Res	Not res
Total (%)	n = 16	7 (44)	9 (56)	10 (63)	6 (36)	11 (69)	5 (31)
Phakic (%)	n = 12	6 (50)	6 (50)	8 (67)	4 (33)	8 (67)	4 (33)
Pseudophakic (%)	n = 4	1 (25)	3 (75)	2 (50)	2 (50)	3 (75)	1 (25)
		p = 0.5692 (NS)		p = 0.604 (NS)		p = 1.000 (NS)	
**OCM**		Res	Not res	Res	Not res	Res	Not res
Total (%)	n = 19	7 (37)	12 (63)	10 (53)	9 (47)	11 (58)	8 (42)
Phakic (%)	n = 15	6 (40)	9 (60)	8 (53)	7 (47)	8 (53)	7 (47)
Pseudophakic (%)	n = 4	1 (25)	3 (75)	2 (50)	2 (50)	3 (75)	1 (25)
		p = 1.00 (NS)		p = 1.00 (NS)		p = 0.6027 (NS)	

OCM, ocular co-morbidity; Res, resolved; Not res, not resolved; NS, non-significant.

P values test the differences between **resolved** phakic vs pseudophakic and the differences between **non-resolved** phakic vs pseudophakic.

Excludes patients with FTMH.

### FTMH resolution

Anatomical closure of the FTMH as confirmed on OCT occurred in four of the six patients at 24 weeks. Rates of resolution were lower at four weeks (33%), however at 12 weeks 50% of patients had resolved, with an increase to four of the six (67%) at 24 weeks. The two ocriplasmin failure cases of FTMH underwent combined phacoemulsification, vitrectomy and macular peel with gas tamponade. The surgeries were both uncomplicated, and the macular hole closed within 2 weeks of the operation.

### Visual acuity

The recorded mean (range) pre-operative visual acuity (logMAR, ETDRS) in the nOCM patient group and in the OCM group were 0.52 and 0.74 respectively (Table 4). Four weeks after treatment, the VA had improved in both groups to 0.42 (nOCM) and 0.43 (OCM). There was a slight deterioration in VA at 12 weeks (see Table 4), which improved at 24 weeks. The VA at 24 weeks was 0.40 in the nOCM group. After 30 weeks in the VMT group with nOCM, the mean LogMAR VA improved from 0.44 to 0.28 (p = 0.0068, paired t-test).

**Table 4:** Visual acuity at 4, 12 and 24 weeks.

	No OCM* (n = 22)	OCM † (n = 25)
Mean VA LogMAR (Snellen)		
4 Weeks	0.42 (6/16)	0.43 (6/16)
12 Weeks	0.51 (6/19)	0.50 (6/19)
24 Weeks	0.40 (6/15)	0.42 (6/16)

OCM, ocular co-morbidity.

*Visual Acuity at Final Follow-up: at mean 30 weeks/7 months.

Overall, mean VA improvement from LogMAR 0.44 to 0.28 for VMT with no OM group (n = 16).

(p = 0.0065, paired t-test).

**†**Visual Acuity at Final Follow-up: at mean 28 weeks/6.5 months.

Overall, mean VA improvement from LogMAR 0.45 to 0.33 for whole VMT group (n = 19).

(p = 0.0158, paired t-test).

Overall, mean VA change from LogMAR 0.74 to 0.75 for FTMH group (n = 6).

(p = 0.8827, paired t-test).

### Safety

There were few adverse events (AEs) reported during the audit period, and all were consistent with expected ocular AEs associated with intraocular injections [10]. In three patients, bilateral photopsia was reported for up to 48 hours post-injection. These symptoms resolved completely without persistent symptoms and VMT release was successful in all three patients. The fellow eyes did not show any abnormality in these patients over the follow-up period.

There were no cases of retinal detachment, and no retinal tears within three months of injection. There were no cases of severe visual loss. There were no instances of new onset or worsening of MH following injection. Two patients who received ocriplasmin for VMT with a FTMH underwent electrophysiological testing for persistent subfoveal fluid at the 12-week visit. There was no significant abnormality in macular or retinal function in either patient.

Seven patients subsequently underwent uncomplicated phacoemulsification and posterior chamber lens implantation, and no zonular problems were reported in these patients.

## Discussion

NICE guidance in the United Kingdom recommends ocriplasmin treatment in the specified subset of patients to be cost effective [9]. All eligible patients in this study were managed as part of the standard care pathway that includes the availability of the following three options: observation alone without any treatment to allow spontaneous resolution; treatment using intravitreal injection of ocriplasmin; or, vitrectomy +/- combined cataract surgery. For patients who elected to receive ocriplasmin, we report up to 69% resolution rate for VMT, and up to 67% macular hole closure rate.

This study highlights the current clinical impact and efficacy of ocriplasmin in VMT and FTMH under NICE guidance. In our hospital eye service, careful patient history, complete ophthalmological review and the use of OCT with overlapping line scans and en-face imaging to exclude any cases with ERM ensure the potential patients meet the strict NICE guidance. The OCT measurement and classification of FTMH was made as per the international Vitreomacular Traction Study Classification System [3]. All eligible patients were given the choice of the following options: observation alone without any treatment, treatment using intravitreal injection of ocriplasmin or surgery as the standard of care. The resolution rates of VMT and FTMH were discussed with patients, including the natural history data, clinical trial data for ocriplasmin, and national benchmarks/outcomes for vitrectomy surgery.

The investigation of the nOCM and OCM groups were undertaken to explore current clinical practice amongst retina specialist at Moorfields. The NICE guidance does not exclude patients with OCM, and there is anecdotal evidence to suggest that patients with OCM may less favourable outcomes following ocriplasmin. In this study VMT release in the OCM group was found to be 37% at the 4-week time point. This compares favourably with results collected from previous studies (MIVI-TRUST trials) investigating the use of ocriplasmin in VMT patients, where the resolution rate was reported as being 26.5% at four weeks [10–12]. In the current study population of carefully selected patients (nOCM), the VMT resolution rate was further increased to 44% at four weeks. This difference in resolution rate highlights the importance of ensuring that patients have the macular conditions correctly classified by OCT, and the advantages of the NICE guidance to maximise patient outcomes.

The resolution in VMT was coupled, as one would expect, with increases in vision measures. LogMAR BCVA was improved from 0.52 at the initial visit to 0.42 at 4 weeks. By 30 weeks, further improvements in vision were seen with the BCVA reaching 0.28. For patients with OCM, the final vision was significantly improved compared to baseline and these effects are similar to the nOCM group. A limitation of this study is the lack of an objective measure/test of metamorphopsia/distortion that can be used for analysis. At present, the change in symptoms of distortion are documented in case records. For all patients with VMT resolution after ocriplasmin, the symptoms reduced following the treatment.

These data can be compared to results previously reported in a study in which the natural history of VMT was assessed [13]. In this study 230 eyes of 185 patients with VMT were followed through observation alone. Spontaneous release of VMT was observed in 73 of the eyes representing a 31.7% resolution rate without treatment but the time to this resolution occurred, on average, 18 months after the initial visit, suggesting a longer time to potential resolution when no intervention is involved. Delays in intervention and observing patients with this disorder have been linked with further deteriorations in the disease progression and thus may not be the most effective management for these patients [14]. In this study 22.7% of patients demonstrated spontaneous release of VMT with a significant proportion of patients exhibiting worsening in the disease progression thus providing more weight that timely intravitreal intervention could benefit those patients presenting with VMT to the ophthalmology clinic to increase the long term prognosis of these patients.

Although the primary efficacy measure for this audit was VMT resolution at four weeks, the resolution rate increased over the period of patient follow up, particularly in the pseudophakic patients (with a resolution rate of 75% at 24 weeks). However, in this audit it is difficult to review differences or provide concrete conclusions on these observations between the sub-populations since numbers were so small.

Since the publication of NICE guidance, the Oasis Study has reported improved efficacy of ocriplasmin for VMT at 41.7% (5), and the results of the phase 4 ORBIT study on real world outcomes are awaited. For the management of VMT, the potential role of intraocular gas tamponade is being increasingly reported with positive successes and larger randomized clinical trials are awaited [15,16].

At Moorfields Eye Hospital, the use of ocriplasmin is administered entirely in accordance with the NICE guidelines. Patients follow a standard pathway for treatment, whereby staff will triage cases as routine referrals, thus allowing a period of observation before the patient arrives in the hospital specialist retinal clinic. The effectiveness of this treatment pathway is reflected in the mean duration of symptoms prior to ocriplasmin injection (23–28 weeks). In this study, it was not possible to look at the relationship between duration of symptoms and VMT resolution, however patients should be promptly treated. NICE recommend that in patients with FTMH (stage II), ocriplasmin should be used during the wait for surgery without delaying the procedure [5]. At MEH all referrals are triaged as routine that are seen in the clinic between 8–10 weeks at the point of referral. The MEH pathway for treatment aims to reduce the delay between referral, diagnosis and treatment. The development of the MEH pathway is the subject of another project, and has helped demonstrate the multidisciplinary approach that should be considered to improve the efficacy of this treatment option to VMT patients.

The number and nature of events reported was within the expectations of the audit team. Photopsia was reported in 3 of the cases. This is a common adverse event that can occur during the treatment of VMT, and in particular may occur when acute vitreous separation take place and as such may have been as a result of the treatment process itself for VMT. There was one case of mild dyschromatopsia, which has been reported as a common adverse reaction in patients injected with ocriplasmin [17] but this resolved fully without further complications and vision improved.

Moorfields have developed clinical pathways for full integration of referral, diagnosis, treatment setting and logistics in order to further optimise the effective treatment using ocriplasmin for VMT at MEH. The numbers of patients with FTMH was low (six) and this reflects the reality of patient choice. Despite a success rate of 67% with ocriplasmin for a medium size FTMH, the current trend for management of patients with a small to-medium FTMH is vitrectomy or phacovitrectomy surgery. The primary reason relates to the very high success rates of MH closure after surgery (90–95%) and this is understandably a more favourable outcome for patients.

Since the introduction of OCT especially in UK community optometric practices, the majority of VMT patients referred to the hospital eye service have asymptomatic VMT. The VMT is detected during routine patient visits for refraction testing at opticians. These patients together with the mild-moderately symptomatic VMT hospital patients opt for the observational management pathway as no intervention is in fact required. This patient group constitutes a significant proportion of patients in our service, and this may be another factor in the low numbers of patients who receive treatment with ocriplasmin in the real world. The NICE guidelines provide excellent guidance for clinicians who manage VMT patients.

## Conclusions

In the real world of post-NICE guidance and ocriplasmin use in a large tertiary referral UK centre, patients appear to be carefully selected for treatment with ocriplasmin in the setting of alternative conservative and surgical management options. We observed better rates of resolution of VMT compared to the landmark clinical trials and recent natural history studies. At Moorfields, the standard practice is currently based on well thought out and planned pathways for the diagnosis and treatment of patients. These pathways reflect the NICE recommendations and are designed in patients’ best interests. The careful diagnosis of patients by use of OCT imaging, and inclusion of only those patients who fit the inclusion criteria for treatment is reflected in the resolution rates observed at four weeks and those improvements seen over longer term follow up.

## Supporting information

S1 Table The raw data for study Part 1. (PDF)

S2 Table The raw data for study Part 2. (PDF)
