# Supplementary material for: Intravitreal ocriplasmin for the treatment of vitreomacular traction and macular hole- A study of efficacy and safety based on NICE guidance
Source: PLoS One. 2018 May 16;13(5):e0197072. doi: 10.1371/journal.pone.0197072 (PMC5955569; doi:10.1371/journal.pone.0197072)
Supplement: S1 Table — (PDF) [file pone.0197072.s001.pdf]

Sex	Lens	Eye	ERM	VMT	VMT width	FTMH size
M	phakic	R	N	Y	451	N
F	phakic	L	N	Y	598	N
F	phakic	R	N	Y	726	N
F	pseudophaki	R	N	Y	505	N
M	phakic	R	N	Y	560	N
F	phakic	R	N	Y	506	N
M	phakic	R	N	Y	697	N
F	pseudophaki	L	N	Y	315	N
F	phakic	L	N	Y	598	N
F	pseudophaki	L	N	Y	816	N
M	phakic	R	N	Y	552	N
F	phakic	R	N	Y	807	N
F	phakic	L	N	Y	380	N
F	pseudophaki	L	N	Y	71	N
F	phakic	R	N	Y	255	N
M	phakic	L	N	Y	207	N
F	PHAKIC	R	N	Y	486	N
F	phakic	L	N	Y	678	N

Sx duration (v	Pre-inj VA	Pre-inj Logma	Pre-inj Date	Pre-inj IOP	JETREA Date	Complication
156	6/24	0.6	23/07/14	16	05/08/14	N
52	6/24	0.6	08/08/14	14	05/09/14	N
48	6/18	0.48	12/03/15		20/03/15	N
26	6/18	0.48	06/06/14	9	13/06/14	N
24	6/24	0.6	14/08/14	12	05/09/14	N
24	6/24	0.6	22/04/14	18	16/05/14	N
24	6/24	0.6	19/08/14	18	04/11/14	N
20	6/24	0.6	25/09/14	10	03/10/14	N
16	6/12	0.3	05/02/15	19	06/02/15	N
12	6/24	0.6	29/01/15	14	06/02/15	N
12	6/24	0.6	20/05/14	20	24/09/14	N
10	6/18	0.48	07/05/15	11	08/05/15	N
8	6/12	0.3	19/01/15	15	03/03/15	N
8	6/9	0.18	16/05/14	13	23/05/14	N
8	6/12	0.3	27/08/15		12/09/15	N
7	6/9	0.18	26/02/15	13	06/03/15	N
	6/24	0.6	12/02/15		06/03/15	N
	6/12	0.3	10/04/15		17/04/15	

Post-op 1 VA	Post-op 1 Da	Post-op 1 An	Post-op 1 IO	Post-op 2 VA	Post-op 2 Da	Post-op 2 An
6/36	13/08/14	VMT	13	6/24	24/09/14	VMT
6/12	12/09/14	VMT	15	6/12	03/10/14	VMT
6/12	26/03/15	VMT		6/12	30/03/16	resolved
6/24	26/06/14	VMT	12	6/24	06/08/14	VMT
6/12	12/09/14	VMT	10	6/24	10/10/14	VMT
6/24	17/06/14	Resolved	21	6/9	23/09/14	Resolved
6/24	16/12/14	VMT	13	6/24	NO DATA	
6/18	10/10/14	Resolved; lar	10	6/12	06/11/14	Resolved; lar
6/18	12/02/15	VMT	14	6/12	02/04/15	Resolved
6/24	12/02/15	VMT	9	6/12	23/04/15	VMT
6/24	28/10/14	VMT	18	6/36	17/02/15	VMT
6/12	14/05/15	VMT		6/12	04/06/15	VMT
6/12	11/03/15	VMT	10	6/12	13/04/15	VMT
6/9	30/05/14	VMT	11	6/6	13/06/14	VMT
	12/11/15	VMT				
6/9	12/03/15	VMT	20	6/9	09/04/15	Resolved
6/24	06/04/15	VMT		6/9	04/06/15	resolved
6/12	23/04/15	RESOLVED		6/9	26/05/15	RESOLVED

Post-op 2 IOI	4 weeks	Post-op 3 VA	Post-op 3 Da	Post-op 3 An	Post-op 3 IOI	Post-op 4 VA
14		6/36	04/03/15	VMT		
15		6/18	12/12/14	VMT	15	
	Y	6_6	04/06/15	resolved		
10		6/24	24/09/14	Resolved	10	6/18
9		6/24				
20	Y	6/18	24/03/15	Resolved	21	
		6/18	10/02/15	Resolved		6/12
14	Y		12/02/15	Resolved	12	
24	Y	6/9	30/04/15	Resolved	19	
13						
14						
14		6/6	11/07/14	VMT no PVD	12	6/9
13	Y					
90	Y					
	Y					

Post-op 4 Da	Post-op 4 An	Post-op 4 IOI	Post-op Logn	follow-up	24 weeks
			0.78	211	
			0.3	98	
			0.3	76	Y
28/01/15	Resolved		0.6	229	Y
			0.6	35	
			0.18	312	Y
26/05/15	Resolved		0.48	203	Y
			0.3	132	Y
			0.18	83	Y
			0.3	76	
			0.78	146	
			0.3	27	
			0.3	41	
15/10/14	Resolved	14	0.18	145	Y
				61	
			0.18	34	Y
			0.18	90	Y
			0.18	39	Y
