# Supplementary material for: Intravitreal ocriplasmin for the treatment of vitreomacular traction and macular hole- A study of efficacy and safety based on NICE guidance
Source: PLoS One. 2018 May 16;13(5):e0197072. doi: 10.1371/journal.pone.0197072 (PMC5955569; doi:10.1371/journal.pone.0197072)
Supplement: S2 Table — (PDF) [file pone.0197072.s002.pdf]

Sex	Lens	Eye	ERM	VMT	VMT width	FTMH size
M	phakic	R	N	Y	697	N
F	phakic	L	N	Y	598	N
F	phakic	R	N	Y	506	N
F	pseudophakic	L	N	Y	71	N
F	phakic	R	N	Y		357
M	phakic	L	N	Y	207	N
F	pseudophakic	R	N	Y	505	N
F	pseudophakic	L	N	Y	816	N
M	phakic	L	N	Y		159
F	phakic	L	N	Y		322
F	phakic	R	N	Y	255	N
M	phakic	R	N	Y	560	N
F	PHAKIC	R	N	Y	486	N
F	phakic	R	N	Y	807	N
F	phakic	R	N	Y	726	N
F	pseudophakic	L	N	Y	315	N
F	phakic	L	N	Y	598	N
M	phakic	R	N	Y		170
F	PHAKIC	R	N	Y		196
M	phakic	R	N	Y		300
F	phakic	L	N	Y	678	

Sx duration (wks)	Pre-inj VA	Pre-inj VA Lo	Pre-inj Date	Pre-inj IOP	JETREA Date	Complication
24	6/24	0.6	19/08/14	18	04/11/14	N
52	6/24	0.6	08/08/14	14	05/09/14	N
24	6/24	0.6	22/04/14	18	16/05/14	N
8	6/9	0.18	16/05/14	13	23/05/14	N
6	6/36	0.78	20/03/14	12	02/05/14	N
7	6/9	0.18	26/02/15	13	06/03/15	N
26	6/18	0.48	06/06/14	9	13/06/14	N
12	6/24	0.6	29/01/15	14	06/02/15	N
9	6/24	0.6	05/03/14	16	01/04/14	N
52	6/60	1	15/05/14	14	23/05/14	N
8	6/12	0.3	27/08/15		12/09/15	N
24	6/24	0.6	14/08/14	12	05/09/14	N
	6/24	0.6	12/02/15		06/03/15	N
10	6/18	0.48	07/05/15	11	08/05/15	N
48	6/18	0.48	12/03/15		20/03/15	N
20	6/24	0.6	25/09/14	10	03/10/14	N
16	6/12	0.3	05/02/15	19	06/02/15	N
12	6/36	0.78	26/02/15		06/03/15	N
60	6/18	0.48	05/02/15		11/02/15	N
12	6/36	0.78	05/03/15		20/03/15	N
	6/12	0.3	10/04/15		17/04/15	

Post-op 1 VA	Post-op 1 Date	Post-op 1 Anatomy	Post-op 1 IOP	Post-op 2 VA	Post-op 2 Dat
6/24	16/12/14	VMT	13	6/24	NO DATA
6/12	12/09/14	VMT	15	6/12	03/10/14
6/24	17/06/14	Resolved	21	6/9	23/09/14
6/9	30/05/14	VMT	11	6/6	13/06/14
6/24	30/05/14	HOLE CLOSED	14	6/24	08/08/14
6/9	12/03/15	VMT	20	6/9	09/04/15
6/24	26/06/14	VMT	12	6/24	06/08/14
6/24	12/02/15	VMT	9	6/12	23/04/15
6/9	30/04/14	HOLE CLOSED	16	6/9	13/08/14
6/36	29/05/14	PVD, FTMH	16	6/60	26/06/14
	12/11/15	VMT			
6/12	12/09/14	VMT	10	6/24	10/10/14
6/24	06/04/15	VMT		6/9	04/06/15
6/12	14/05/15	VMT		6/12	04/06/15
6/12	26/03/15	VMT		6/12	30/03/16
6/18	10/10/14	Resolved; lamellar cl	10	6/12	06/11/14
6/18	12/02/15	VMT	14	6/12	02/04/15
6/36	12/03/15	FTMH, VMT	10	6/36	16/04/15
6/18	26/02/15	FTMH			
6/36	26/03/15	FTMH		6/36	04/06/15
6/12	23/04/15	RESOLVED		6/9	26/05/15

Post-op 2 Anatomy	Post-op 2 IOF	**4 weeks**	Post-op 3 VA	Post-op 3 Dat	Post-op 3 Ana
			6/18	10/02/15	Resolved
VMT	15		6/18	12/12/14	VMT
Resolved	20	Y	6/18	24/03/15	Resolved
VMT	14		6/6	11/07/14	VMT no PVD
Resolved		Y	6/24	05/12/14	Resolved
Resolved	13	Y			
VMT	10		6/24	24/09/14	Resolved
VMT	13				
HOLE CLOSED	14	Y			
PVD, FTMH	16		CF	04/09/14	PVD, FTMH
VMT	9		6/24		
resolved		Y			
VMT					
resolved		Y	6/6	04/06/15	resolved
Resolved; lamellar chan	14	Y		12/02/15	Resolved
Resolved	24	Y	6/9	30/04/15	Resolved
FTMH, VMT	11		6/24	11/06/15	closed
HOLE CLOSED		Y			
RESOLVED		Y			

Post-op 3 IOF	Post-op 4 VA	Post-op 4 Dat	Post-op 4 An	Post-op 4 IOF	Post-op VA L	follow-up
	6/12	26/05/15	Resolved		0.48	203
15					0.3	98
21					0.18	312
12	6/9	15/10/14	Resolved	14	0.18	145
17					0.6	217
					0.18	34
10	6/18	28/01/15	Resolved		0.6	229
					0.3	76
					0.18	134
					2	104
						61
					0.6	35
					0.18	90
					0.3	27
					0	76
12					0.3	132
19					0.18	83
12					0.78	97
					0.6	15
					0.78	76
					0.18	39

24 weeks
Y
Y
Y
Y
Y
Y
Y
Y
Y
Y
Y
Y
Y
Y
